# Deep Response in Multiple Myeloma: A Critical Review

**DOI:** 10.1155/2015/832049

**Published:** 2015-12-10

**Authors:** Mariateresa Fulciniti, Nikhil C. Munshi, Joaquin Martinez-Lopez

**Affiliations:** ^1^Jerome Lipper Multiple Myeloma Center, Dana-Farber Cancer Institute, Harvard Medical School, Boston, MA 02215, USA; ^2^Department of hematology, Hospital 12 de Octubre, Unidad CRIS, CNIO, University of Madrid, Spain

## Abstract

Novel and more effective treatment strategies against multiple myeloma (MM) have significantly prolonged patients' survival and raised interest in the depth of response and its association with clinical outcome. Minimal residual disease (MRD) has emerged as one of the most relevant prognostic factors in MM and should be included in a new definition of complete response (CR). Although further standardization is still required, MRD monitoring should be applied in prospective clinical trials as a sensitive tool to compare and evaluate the efficacy of different treatment strategies, particularly in the consolidation and maintenance settings, and implement individualized therapy-monitoring approaches. Here, we review current definition of deep response in MM, advantages and limitations of current MRD assessment assays, clinical evidences for MRD monitoring as a prognostic tool for therapeutic decisions in MM, and challenges to develop uniform criteria for MRD monitoring.

## 1. Introduction

Multiple myeloma is a complex disease characterized by the presence of profound intratumoral heterogeneity that increases progressively from the stages of monoclonal gammopathy of undetermined significance (MGUS) and asymptomatic MM to symptomatic or clinical MM [1–3]. The introduction of novel therapies for the treatment of multiple myeloma (MM) patients has significantly improved clinical outcome [4]; however, majority of the patients relapse, making myeloma still an incurable disease [5, 6]. The challenge now is to identify the population of patients with aggressive disease and therefore poor prognosis [7, 8]. Although the ideal way to classify patients with different prognosis is at diagnosis, usually it is extremely difficult, and therefore response monitoring is becoming more relevant in MM.

Complete response (CR), defined by negative immunofixation (IFX) and less than 5% bone marrow plasma cells, has been accepted as a relevant surrogate marker of survival [9]. This definition of clinical response criteria and clinical end points has largely remained the same over the past 15 years [8, 10–12] and presents several relevant limitations [8, 13]. The challenge is to identify the patients that despite reaching CR status relapse very quickly (unsustained response) compared to other patients that only achieve partial response but have prolonged survival.

As CR rates have improved, more rigorous definitions of response have been developed. In the last consensus criteria of response in MM, three new concepts have been incorporated: stringent CR (sCR), immunophenotypic CR (iCR), and molecular CR (mCR) (Table 1). These deep response criteria are all based on different methodologies and provide discordant results [14–25] making the scenario very confusing. Importantly, published data show that establishing some levels of deep response in MM could translate in different prognosis impact: patients achieving grade CR^3^ (0,1% deep complete response) had a projected progression-free survival of 35–45 months, while patients achieving CR^5^ (0.001% deep complete response grade) had a projected progression-free survival of more than 80 months [26, 27] (Table 2). These levels of disease reduction have prognosis impact, independently of the techniques employed.

A growing body of evidence demonstrates that detection of subclinical levels of myeloma (i.e., minimal residual disease, MRD) provides powerful independent prognostic information [23], and categories defining deep response should be updated according to the levels of MRD. Chronic myeloid leukemia (CML) is the first disease in which this approach was applied to normalize the criteria for a deep response [28, 29]. Consequently, there is an increasing interest in the use of MRD detection to provide early end points in clinical trials and to inform myeloma patient management.

Therefore, a new definition of CR including different levels of MRD is needed in MM to compare different treatment strategies and develop a truly personalized approach to MM therapy. Likewise, this definition will be applied in all clinical settings and will be interchangeable between different centers.

## 2. Methodologies for Assessing Minimal Residual Disease in Myeloma

Improving CR rates have made the measurement and monitoring of MRD in MM a relevant task. However, implementation of MRD assessment into clinical practice is a major challenge, hampered by differences in the assays and analytical methods employed between different routine laboratories. Most patients who achieve MRD-negative status eventually relapse, indicating that the sensitivity and specificity of traditional techniques for MRD assessment can be improved. Recent data by Rawstron et al. [27] suggests that a lower cutoff provided by more sensitive assays (e.g., next generation sequencing (NGS) or high-sensitive multiparameter flow cytometry (MFC)) will likely improve outcome prediction further. This has already been confirmed by Martinez-Lopez et al. using NGS [26] who identified 3 groups of patients with different time to progression (TTP): patients with high (<10^−3^), intermediate (10^−3^ to 10^−5^), and low (>10^−5^) MRD levels showed significantly different TTP (27, 48, and 80 months, resp.). Accordingly, 10^−5^ should currently be considered as the target cutoff level for definition of MRD negativity.

In this section, we will consider the various methodologies available at present for MRD detection, taking account of their relative advantages and limitations.

### 2.1. Serologic Methods, to Determine the Tumor Production

Measurements of monoclonal protein biomarkers, which can be in the form of intact immunoglobulin, immunoglobulin fragments, or free immunoglobulin light chains (FLC), in either the serum or the urine are all widely available and noninvasive methods used for diagnosis and monitoring of disease burden in MM. One of the earliest identifıed biomarkers is Bence Jones protein, described in 1848 [30].

During the past decade the measurement of serum kappa and lambda free light chains (sFLCs) has also become part of routine clinical testing, particularly for the diagnosis and follow-up of patients with nonsecretory and oligosecretory myeloma, light chain myeloma, and amyloidosis [31].

The International Myeloma Working Group (IMWG) in 2006 has introduced normalization of sFLCs and absence of clonal PCs in BM biopsies by immunohistochemistry and/or immunofluorescence as additional requirements to define more stringent CR criteria [10]. sFLC ratio has been shown at diagnosis to be an independent prognostic factor and predict more aggressive disease [32] with potential to improve risk stratification as well [33]. However, several other studies show contradictory results [31, 34, 35], even with regard to response [36, 37], and it remains controversial how to incorporate sFLCs measurement into MRD monitoring in MM. These studies found that normalization of sFLCs was not associated with increased survival in patients in conventional CR. In addition, it has been suggested that the sFLC might be replaced by the heavy-light format and become merely a surrogate for recovery of the immune system rather than MRD monitoring tool. Therefore, in our opinion, sFLCs should not be considered as a method for MRD assessment in myeloma.

The US Food and Drug Administration- (FDA-) approved heavy/light chain (HLC) assay (Hevylite) measures suppression of the uninvolved HLC pair (e.g., IgG-lambda, IgA-kappa, and IgA-lambda for a patient with IgG-kappa disease). The HLC ratio reflects the balance between monoclonal and polyclonal immunoglobulins of involved and uninvolved isotypes taking into account the polyclonal plasma cell suppression or expansion that occurs with the treatment. However, only few studies have shown the ability of the Hevylite assay to give additional prognostic information in MGUS and MM [38]. In general, the assay does not add any value to immunofixation or sFLC tests, although it could have some advantages in monitoring patients with M component migrating in *β* regions [39].

### 2.2. Bone Marrow (BM) Methodologies to Determine the Tumor Burden

Morphologic bone marrow examination is one of the most commonly used methods to measure tumor burden in MM. Two different studies demonstrate that microscopic assessment of the BM can have prognostic value [40, 41]. However, the sensitivity of morphology alone is limited by the number of cells evaluated as well as sampling variability. Moreover, BM biopsies are expensive and invasive, posing some risk to patients.

Multiparameter flow cytometry (MFC) and Ig allele-specific oligonucleotide-based quantitative polymerase chain reaction (ASO-PCR) have emerged as the most attractive, well-suited, and sensitive approaches to detect MRD in the BM of MM patients during and after therapy [35]. Molecular monitoring of disease by MFC and PCR has been commonly used in chronic myelogenous leukemia, acute lymphoblastic leukemia, and acute promyelocytic leukemia to help determine prognosis and guide therapy [42–44].

In myeloma, several reports have demonstrated the ability of both MFC and quantitative PCR to stratify patient cohorts with different prognoses [21, 25, 45]. However, both techniques have some disadvantages and neither has become a standard of care in MM. ASO-PCR in MM is associated with high technical complexity and low applicability [46]. MFC has a higher applicability, virtually covering all patients without requiring patient-specific diagnostic phenotypic profiles [21, 22, 25, 47]. In recent years, the sensitivity of MFC has increased (between 10^−4^ and 10^−5^) because of simultaneous assessment of ≥8 markers in a single tube that can readily identify aberrant PC phenotypes at MRD levels if sufficient cell numbers (e.g., ≥5 × 10^6^) are evaluated [47]. The requirement for extensive expertise in MFC analysis and the lack of a well-standardized flow-MRD method are important disadvantages of MFC immunophenotyping. Additionally, no tumor cells are detectable by MFC or PCR in a fraction of patients who ultimately relapse, indicating that further improvement and standardization efforts are required. Recent reports have demonstrated the utility of high-throughput sequencing- (HTS-) based MRD assessment in lymphoid malignancies [26, 48]. This quantitative method, termed the LymphoSIGHT platform, relies on consensus primers to universally amplify and sequence all rearranged immunoglobulin gene segments present in a myeloma clone. Preliminary studies have shown that NGS of Ig genes might be applicable for MRD detection in BM of MM patients [8]. The sequencing method demonstrated applicability higher than 90% and assay sensitivity ≤10^−6^, with the potential to be distributed across multiple laboratories, because it relies on automated data analysis and does not involve expert interpretation by an operator. Moreover, molecular techniques are not influenced by genetic heterogeneity and clonal tiding throughout patients' treatment. However, additional validations are needed to prove and confirm the utility of this technology for patient risk stratification.

### 2.3. Imaging Techniques

Unlike other hematologic disorders such as acute leukemia, the pattern of BM infiltration in MM is not uniform. Moreover, hemodiluted BM aspirates may lead to false-negative results. These aspects, together with extramedullary disease, represent a potential challenge and pitfall common to all techniques that use BM samples for MRD assessment, as nonrepresentative samples of disease infiltration are sometimes obtained. For this reason, MRD-negative results may correspond to a false-negative case. The use of alternative methods for disease assessment such as imaging techniques [24, 49], monitoring of clonogenic MM progenitors [49, 50], or MM circulating tumor cells [51] could provide complementary information to MRD and improve the estimation of the risk of progression.

Multiple myeloma presents a high frequency of extramedullary relapses, and sensitive imaging techniques have become relevant in assessing low levels of disease outside BM. Magnetic resonance imaging (MRI) is the most sensitive noninvasive imaging technique for detection of bone involvement in the spine and also provides relevant information on the extent and nature of soft tissue disease and the pattern of marrow infiltration (normal, focal, heterogeneous, or diffuse). However, due to treatment-induced necrosis and inflammation, focal lesions may remain hyperintense in both responding and nonresponding patients for several months after therapy, making MRI-based CR inconsistent. While MRI does not properly identify myeloma active lesions after treatment, imaging by fluorodeoxyglucose-positron emission tomography (PET) has been shown to have prognostic significance [24, 52] and would represent the most effective imaging tool to monitor MRD in MM. A specific advantage of PET imaging relies on its ability to detect extramedullary disease, which represents an adverse prognostic event. PET imaging is widely available; however it has some major issues: not all MM patients have PET-avid lesions and interpretation of data can be a challenge considering heterogeneity of visual criteria and poor interobserver reproducibility. Therefore, standardization of response definitions by PET as well as comparison with other sensitive BM-based MRD methods is needed to implement this imaging technique across different clinical studies.

## 3. Standardization and Harmonization

MRD monitoring variability between different clinical laboratories is a major challenge. Because of the prognostic value of MRD in MM, a key goal of the standardization effort is to eliminate or correct the relative differences between MRD negativity assessment and response rates across laboratories.

Optimal use of clinical guidelines for disease diagnosis and patient management requires first standardization and then harmonization, to maximize compatibility, interoperability, safety, repeatability, and quality as well as achieve uniformity of results [53, 54]. Results that are neither standardized nor harmonized may lead to erroneous clinical, financial, regulatory, or technical decisions. While some initiatives on standardization have been performed in chronic myeloid leukemia on molecular MRD [29], there is a lack of standardization and harmonization for MRD assessment by flow cytometry [47]. Different MFC groups need to adopt standardized and validated antibody panels, sample processing, and cell-analysis methods such as those developed by the EuroFlow consortium for MFC, in order to become a universal and fully standardized option for MRD assessment. Standardization of flow cytometric and molecular MRD testing is vital to ensure better and uniform assessment of response and clinical prognostication.

We here propose a roadmap for standardization of MRD assessment in MM:

1. Development of reference standard and referral material to define CR grades 3 and 5.
2. Manufacturing internal calibrators.
3. Evaluation of current degree of measurement equivalence.

MRD assessment in MM based on test results from a specific clinical laboratory measurement procedure (CLMP) without considering the possibility or likelihood of differences between various CLMPs should be flawed. When this happens, aggregation of data from different research clinical trials and development of appropriate clinical practice guidelines will be flawed by the lack of standardized or harmonized results.

Previous experiences of standardization and harmonization of molecular techniques have been difficult and not very well accepted for many laboratories; furthermore the implementation of standardization and harmonization has been hard and long. However, when application in real clinical practice has started the results have been positive. In MM, standardization and harmonization of MRD assessment techniques should be considered, especially if these techniques will be considered biomarkers of response in clinical trials and in the regular clinical practice.

## 4. MRD Assessment in Myeloma: Clinical Applications

Intra- and interpatient heterogeneity in multiple myeloma underscore the need for personalized treatment approaches. In an era of increased treatment options, there are substantial data showing the association of depth of response and clinical outcome. Achievement of CR is considered one of the strongest prognostic biomarkers in MM, both in the transplant and nontransplant settings, although the sCR criteria have failed to unequivocally demonstrate superior prognostic value compared with CR [37].

### 4.1. MRD Adapted Treatments

MRD assessment is a relevant concept in myeloma and several studies using different MRD techniques have shown its value for evaluation of the efficacy of specific treatment stages and, therefore, potential treatment decisions. Overall, persistence of MRD is always an adverse prognostic feature, even among CR patients, but, so far, no clinical trial has randomized MM patients according to their MRD status and, thereby, investigated the role of MRD for individualized therapy. Achievement of MRD negativity may ultimately serve as a primary end point in clinical trials for MM and should be included in the CR criteria. However, patients achieving MRD negativity eventually relapse, and at this point we still do not know if these patients should receive the full-programmed treatment besides reaching the MRD negativity status or asymptomatic relapse. Importantly, considering the patchy pattern of BM infiltration observed in MM that leads to a degree of ambiguity regarding MRD-negative results, it would be safer to make clinical decisions based on MRD positivity rather than on MRD negativity. Several other questions on how to incorporate MRD evaluation in the treatment strategy for MM patients remain to be answered. Can we decide which patients should receive consolidation therapy based on MRD measurement? Can MRD monitoring be used to determine the need for or duration of maintenance therapy? How does maintenance therapies modify MRD levels?

### 4.2. Timing of MRD Evaluation

An important aspect to be considered is the time of MRD monitoring during the course of the treatment. Most studies have been carried out after transplantation in younger patients and after induction in elderly patients, but it is still unclear when the best time is to measure and integrate MRD measurement into therapeutic decision-making.

### 4.3. MRD Kinetics

Changing of MRD levels over time (MRD kinetics) could be relevant for a better evaluation of MM patients and needs further evaluation. For example, both the Spanish and the United Kingdom study groups have shown that MRD kinetics before and after HDT/ASCT allow the identification of chemosensitive (MRD-negative cases at 2 time points), intermediate, and chemoresistant patients (MRD-positive patients at 2 time points). A small clinical study of the Italian group showed three patterns of kinetics: high tumor burden, low tumor burden, and active disease, which could predict the relapse [55]. These clinical studies suggest that MRD kinetics are more informative than single time point assessments and may be useful to address specific clinical questions (e.g., early versus delayed HDT/ASCT for CR patients after induction). Therefore, additional studies should be performed in this regard to avoid overtreatment and undertreatment, particularly during consolidation and maintenance.

### 4.4. MRD Detection Methods

Finally, sensitive methods of MRD detection (1 in 10^5^cells) may contribute to the design of patient-specific treatment approaches. Extensive research is still warranted to determine how to best integrate medullary and extramedullary MRD monitoring, and a process of standardization and harmonization of these methodologies is required. Harmonized MRD approaches not only will provide backwards compatibility with established assays but will also offer sufficiently high enough sensitivity as treatment strategies evolve to remain relevant for the next decade.

*In summary*, (i) MRD assessment is ready for clinical application as biomarker to evaluate the response to different therapies in MM; (ii) MRD could be used to measure the efficacy of different treatments in clinical trials; (iii) MFC and NGS are both equally valid for MRD assessment in MM; (iv) standardization and harmonization are the next steps for MRD assessment in MM.

## 5. Conclusions

Improvement in MM patient outcomes can be achieved through adaptive clinical trials involving risk models based on multiple biomarkers, but several questions are still unanswered. Different clinical trials integrating these approaches to confirm the clinical benefits of MRD monitoring are currently ongoing in myeloma and will hopefully provide the rationale for the use of MRD assessment in the evolving MM clinical paradigm. Moreover, the new generation of biomarkers, including epigenetics, novel imaging, clone burden, GEP signature, and next generation sequencing, coupled with established prognostic biomarkers holds promise for improved stratification of patients with myeloma into specific therapies and clinical trials.

## Figures and Tables

**Table 1:** Definition of response according to the last classification of the IMF.

Categories of response according to IMF 2011	Level of detection
PR	MC < 50%
VGPR	MC < 90%
nCR	MC 0.1–0.5 g/dl (EF−/IF+)
CR	MC ≤ 0,5 g/dl (IF−)
	PC in BM < 5%
Stringent CR	sFLC ratio +BM ICH
Immunophenotypic CR	sCR+ nonaberrant PC in 1,000,000 cells
Molecular CR	CR+ nonclonal plasma cells with sensitivity > 10^−5^

**Table 2:** Proposed new definition for deep response in multiple myeloma.

New proposal for deep response	Level of detection	Project PFS
Deep CR grade 3 CR^3^	Nonclonal plasma cells below 10^−3^, highly sensitive techniques FCM or sequencing should be employed	>35–45 months

Deep CR grade 5 CR^5^	Nonclonal plasma cells below 10^−5^, highly sensitive techniques FCM or sequencing should be employed	>80 months

PR, partial response; MC, monoclonal component; PC, plasma cell; CR complete response; SFLC, serum free light chain; BM, bone marrow.
